# A workflow for the automatic segmentation of organelles in electron microscopy image stacks

**DOI:** 10.3389/fnana.2014.00126

**Published:** 2014-11-07

**Authors:** Alex J. Perez, Mojtaba Seyedhosseini, Thomas J. Deerinck, Eric A. Bushong, Satchidananda Panda, Tolga Tasdizen, Mark H. Ellisman

**Affiliations:** ^1^Center for Research in Biological Systems, National Center for Microscopy and Imaging Research, University of CaliforniaSan Diego, La Jolla, CA, USA; ^2^Department of Bioengineering, University of CaliforniaSan Diego, La Jolla, CA, USA; ^3^Scientific Computing and Imaging Institute, University of UtahSalt Lake City, UT, USA; ^4^Regulatory Biology Laboratory, Salk Institute for Biological StudiesLa Jolla, CA, USA; ^5^Department of Neurosciences, University of CaliforniaSan Diego, La Jolla, CA, USA

**Keywords:** serial block-face scanning electron microscopy, 3D electron microscopy, electron microscopy, automatic segmentation, image processing, organelle morphology, neuroinformatics

## Abstract

Electron microscopy (EM) facilitates analysis of the form, distribution, and functional status of key organelle systems in various pathological processes, including those associated with neurodegenerative disease. Such EM data often provide important new insights into the underlying disease mechanisms. The development of more accurate and efficient methods to quantify changes in subcellular microanatomy has already proven key to understanding the pathogenesis of Parkinson's and Alzheimer's diseases, as well as glaucoma. While our ability to acquire large volumes of 3D EM data is progressing rapidly, more advanced analysis tools are needed to assist in measuring precise three-dimensional morphologies of organelles within data sets that can include hundreds to thousands of whole cells. Although new imaging instrument throughputs can exceed teravoxels of data per day, image segmentation and analysis remain significant bottlenecks to achieving quantitative descriptions of whole cell structural organellomes. Here, we present a novel method for the automatic segmentation of organelles in 3D EM image stacks. Segmentations are generated using only 2D image information, making the method suitable for anisotropic imaging techniques such as serial block-face scanning electron microscopy (SBEM). Additionally, no assumptions about 3D organelle morphology are made, ensuring the method can be easily expanded to any number of structurally and functionally diverse organelles. Following the presentation of our algorithm, we validate its performance by assessing the segmentation accuracy of different organelle targets in an example SBEM dataset and demonstrate that it can be efficiently parallelized on supercomputing resources, resulting in a dramatic reduction in runtime.

## Introduction

**Figure 1:**
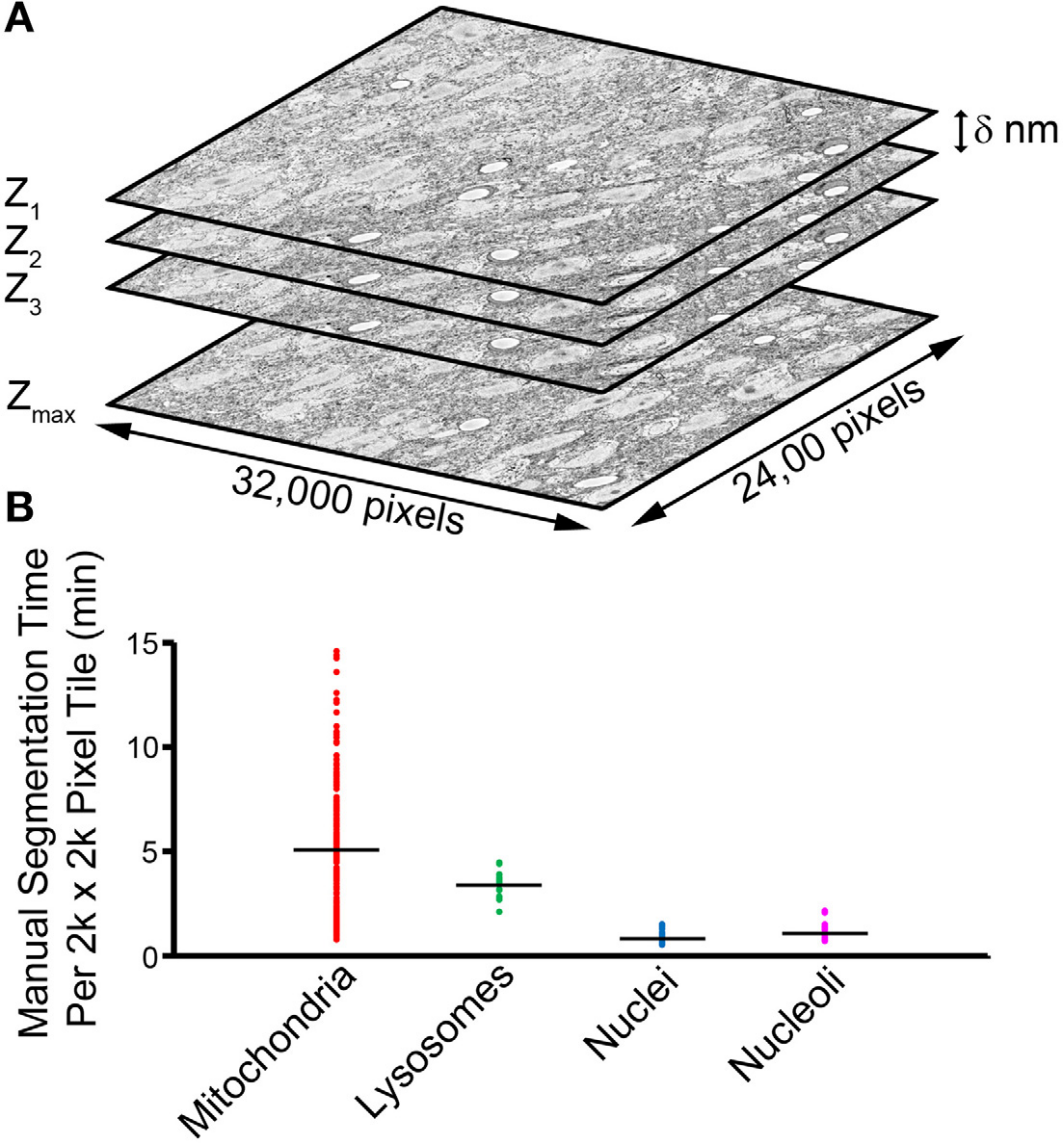
**The manual segmentation of organelles from SBEM image stacks represents a significant bottleneck to quantitative analyses**. **(A)** A typical SBEM dataset consists of individual image slices collected in increments of δ nm, with the values of δ reported in the literature typically falling in the range of 20–100 nm (Peddie and Collinson, 2014). To cover a neuroanatomical region of any significance, the size of such datasets quickly enters the realm of teravoxels and analyses utilizing manual segmentation become intractable. **(B)** A scatter plot of the amount of time required for a highly trained neuroanatomist to segment all instances of a specific organelle in SBEM tiles of size 2000 × 2000 pixels demonstrates this impediment. Average values are represented by horizontal bars (mitochondria = 5.01 min, lysosomes = 3.43 min, nuclei = 0.93 min, nucleoli = 1.24 min). Since mitochondria are ubiquitously present throughout most tissues, extrapolation of their average segmentation time per tile to the size of a full dataset can reliably predict the actual segmentation time required for such a volume. For a dataset the size of the one used in this report (stack volume ~450,000 μm^3^, tile size ~60 μm^2^), the manual segmentation of all mitochondria would require roughly 2.3 years, placing it well outside the realm of feasibility. This effect is further exacerbated when experiments requiring segmentations from SBEM stacks over multiple samples or experimental conditions are desired.

Advances in instrumentation for 3D EM are fueling a renaissance in the study of quantitative neuroanatomy (Peddie and Collinson, 2014). Data obtained from techniques such as SBEM (Denk and Horstmann, 2004) provide unprecedented volumetric snapshots of the *in situ* biological organization of the mammalian brain across a multitude of scales (Figure 1A). When combined with breakthroughs in specimen preparation (Deerinck et al., 2010), such datasets reveal not only a complete view of the membrane topography of cells and organelles, but also the location of cytoskeletal elements, synaptic vesicles, and certain macromolecular complexes.

Harnessing the power of these emerging 3D techniques to study the structure of whole cell organellomes is of critical importance to the field of neuroscience. Abnormal organelle morphologies and distributions within cells of the nervous system are characteristic phenotypes of a growing number of neurodegenerative diseases. Aberrant mitochondrial fragmentation is believed to be an early and key event in neurodegeneration (Knott et al., 2008; Campello and Scorrano, 2010), and changes in mitochondrial structure have been observed in Alzheimer's disease (AD) neurons from human biopsies (Hirai et al., 2001; Zhu et al., 2013). Additionally, altered nuclear or nucleolar morphologies have been observed in a host of pathologies, including AD (Mann et al., 1985; Riudavets et al., 2007), torsion dystonia, (Kim et al., 2010), and Lewy body dementia (Gagyi et al., 2012).

Our ability to quantify and understand the details of these subcellular components within the context of large-scale 3D EM datasets is dependent upon advances in the accuracy, throughput, and robustness of automatic segmentation routines. Although a number of studies have extracted organelle morphologies from SBEM datasets via manual segmentation, (Zhuravleva et al., 2012; Herms et al., 2013; Holcomb et al., 2013; Wilke et al., 2013; Bohórquez et al., 2014), their applications are limited to only small subsets of the full stack due to the notoriously high labor cost associated with manual segmentation (Figure 1B). Automatic segmentations generated based on thresholds or manipulations of the image histogram (Jaume et al., 2012; Vihinen et al., 2013) may require extensive manual editing of their results to achieve the accurate quantification of single organelle morphologies.

The development of computationally advanced methods for the automatic segmentation of organelles in 3D EM stacks has led to increasingly accurate results (Vitaladevuni et al., 2008; Narashima et al., 2009; Smith et al., 2009; Kumar et al., 2010; Seyedhosseini et al., 2013a). Recently, Giuly and co-workers proposed a method to segment mitochondria utilizing patch classification followed by isocontour pair classification and level sets (Giuly et al., 2012). Lucchi et al. (2010, 2012) developed an approach that trains a classifier to detect supervoxels that are most likely to belong to the boundary of the desired organelle. An approach to automatically segment cell nuclei using the software package ilastik to train a Random forest voxel classifier followed by morphological post-processing and object classification was proposed by Sommer et al. (2011), Tek et al. (2014). Though they yield impressive results, many current approaches utilize assumptions about the 3D morphology of the organelle target. This is problematic not only because it makes their expansion to the segmentation of other organelles non-trivial, but also because the typical SBEM dataset contains a heterogeneous mixture of organelle morphologies across multiple cell types. Therefore, there is a clear need for a robust method to accurately segment various organelles in SBEM stacks without any *a priori* assumptions about organelle morphology.

In this work, we present a method for the robust and accurate automatic segmentation of morphologically and functionally diverse organelles in EM image stacks. Organelle-specific pixel classifiers are trained using the cascaded hierarchical model (CHM), a state-of-the-art, supervised, multi-resolution framework for image segmentation that utilizes only 2D image information (Seyedhosseini et al., 2013b). A series of tunable 2D filters are then applied to generate accurate segmentations from the outputs of pixel classification. In the final processing step, 3D connected components are meshed together in a manner that minimizes the deleterious effects of local and global imaging artifacts. Finally, we demonstrate that our method can be easily and efficiently scaled-up to handle the segmentation of all organelles in teravoxel-sized 3DEM datasets.

## Material and methods

The description and validation of our method are arranged into three sections. In the first section, the workflow is described in detail. In the second, the robustness and accuracy of our method are validated by applying it to four different organelle targets (mitochondria, lysosomes, nuclei, and nucleoli) from a test SBEM dataset. In the third section, we describe experiments that demonstrate how our method can be easily scaled-up to accommodate the segmentation of teravoxel-sized datasets.

### The proposed method

#### Image alignment and histogram specification

All individual images of the input SBEM stack are converted to the MRC format and appended to an 8-bit MRC stack using the IMOD programs *dm2mrc* and *newstack*, respectively (Kremer et al., 1996). Sequential images within the stack are then translationally aligned to one another in the XY-plane using the cross-correlational alignment algorithm of the IMOD program *tiltxcorr*. To ensure consistency throughout the stack, the histograms of all images are matched to that of the first image in the stack using a MATLAB (The MathWorks, Inc., Natick, MA, U.S.A.) implementation of the exact histogram specification algorithm (Coltuc et al., 2006).

#### Generation of training images and labels

Once an organelle target has been selected by the experimenter, the next step is to generate a set of organelle-specific training images and labels to subsequently train a CHM pixel classifier. A set of N seed points, P, are selected throughout the processed SBEM stack in locations that possess at least one instance of the desired organelle, such that:

$${\text{P}}_{\text{i}}=({\text{x}}_{\text{i}},{\text{y}}_{\text{i}},{\text{z}}_{\text{i}})\forall \text{i}\in \{1,\ldots ,\text{N}\}$$

**Figure 2:**
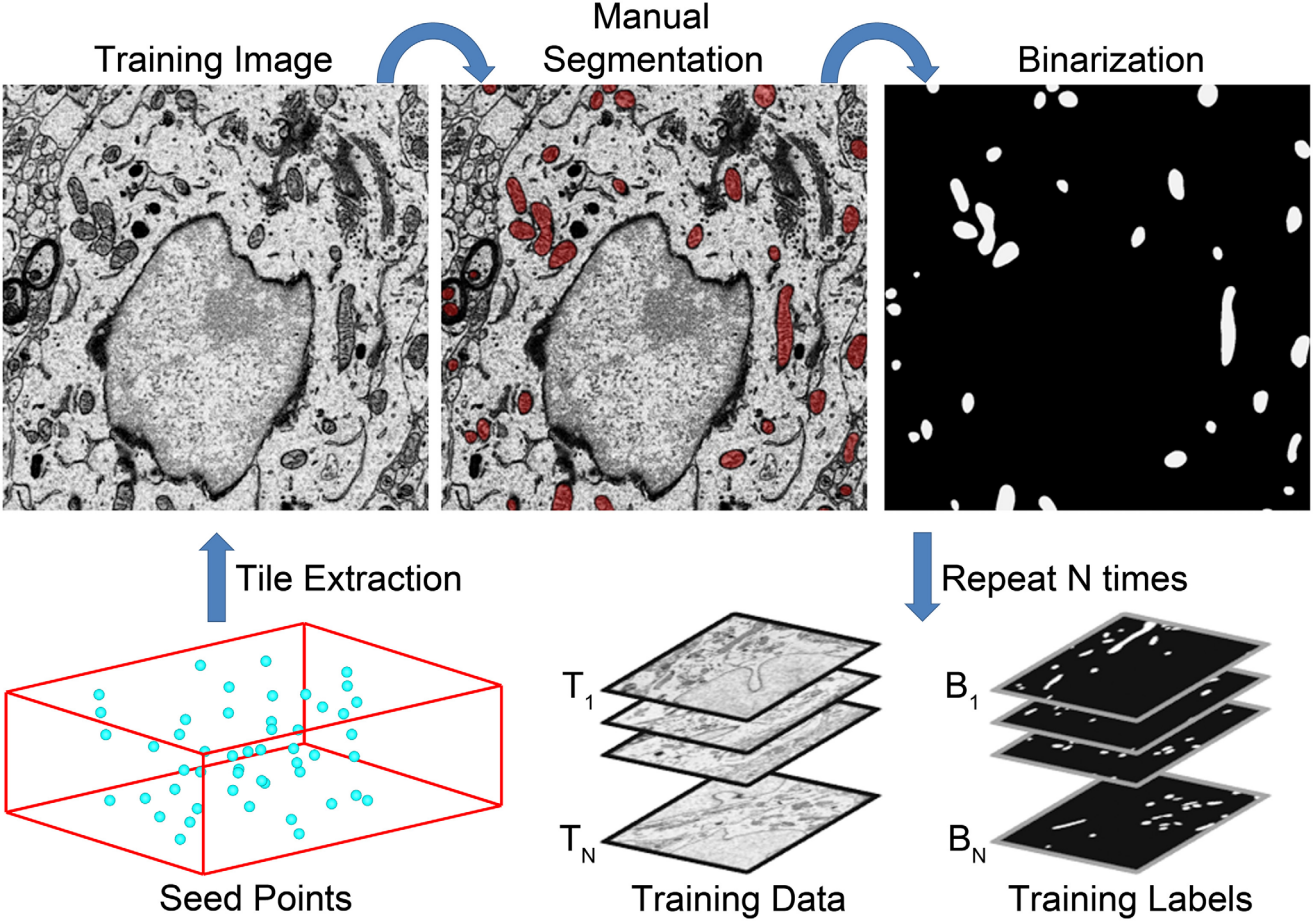
**A flow chart of the steps involved in training data generation**. The generation of a set of training data for mitochondrial automatic segmentation is shown here. First, a set of seed points, P_i_, are selected such that a wide distribution throughout the volume is achieved (bottom left). Tiles of size Q × R centered at each seed point are extracted to serve as training images, T_i_. All instances of the desired organelle target are manually segmented by a trained neuroanatomist on each training image. These manual segmentations are then used as masks to binarize each T_i_ such that pixels of value one correspond to pixels of T_i_ that are positive for the desired organelle. This process is repeated N times to yield stacks of training images and their corresponding training labels, B_i_. These stacks are then used to train a CHM classifier, C_S,L_, with the desired number of stages, S, and levels, L.

These points should be chosen in a manner that yields a wide distribution throughout the stack. After the selection of seed points, every instance of the chosen organelle is manually segmented in a Q × R pixel tile centered at each P_i_. Following manual segmentation, all tiles are extracted from the full SBEM stack using the IMOD program *boxstartend*. The extracted tiles will serve as training images, T_i_. Binary training labels, B_i_, are generated from each T_i_ by applying the corresponding manual segmentation as a mask using the IMOD program *imodmop*. Thus, the final outputs from training data generation are (1) a stack of 8-bit, grayscale training images, T_i_, and (2) a stack of corresponding binary organelle masks, B_i_. Both stacks are of size Q × R × N. A flow chart illustrating this process is shown in Figure 2.

#### Training organelle pixel classifiers with the cascaded hierarchical model

The CHM consists of bottom-up and top-down steps cascaded in multiple stages (Seyedhosseini et al., 2013b). The bottom-up step occurs in a user-specified number of hierarchical levels, L. At each level, the input stacks T_i_ and B_i_ are sequentially downsampled and a classifier is trained based on features extracted from the downsampled data as well as information from all lower levels of the hierarchy. After classifiers have been trained at all levels, the top-down path combines the coarse contextual information from higher levels into a single classifier that is applicable to images at native resolution. This whole process is then cascaded in a number of stages, S, where the output classifier from the previous stage serves as the input classifier for the subsequent stage. The final output is a pixel classifier, C_S,L_, that is applicable to images at the native pixel size of T_i_ and B_i_. For optimal results, the number of stages chosen should be greater than one. The exact number of stages and levels chosen depends on a host of factors, including the size of T_i_ and B_i_ and the computational resources available to the experimenter.

#### Probability map generation

In the next step, a stack of test images, I_j_, are selected to apply the pixel classifier to. Depending on the goals of the experiment, these images may be full slices of the SBEM volume or extracted subvolumes. Prior to pixel classification, each I_j_ is split into an *m × n* array of tiles such that the dimensions of each tile are roughly equivalent to the lateral dimensions of the training stacks, Q × R (step 3 of Algorithm 1). Tiling is performed with an overlap of U pixels between adjacent tiles. The choice of U is dependent on the size of the training stacks as well as the organelle target; in general, ideal values of U should fall in the range of 2–10% of Q and R. The previously generated CHM pixel classifier, C_S,L_, is then applied to each tile, yielding *m × n* probability map tiles (step 5 of Algorithm 1). All processed tiles are then stitched together to yield a final probability map, M_j_ (step 7 of Algorithm 1). When stitching, the pixels in M_j_ that correspond to regions of overlap between adjacent tiles are set to the maximum intensity pixel from all contributing tiles. Finally, M_j_ is normalized such that each pixel ranges from [0, 1], with one representing the highest probability (step 8 of Algorithm 1). This process is then repeated over each I_j_ to yield the final stack of probability maps.

**Algorithm 1:** **Organelle segmentation using tiled input images**.

1:	Declare values of m, n, U, G, α, and λ
2:	**for** every test image I_j_ **do**
3:	Generate k = m × n tiles of I_j_ with overlap U
4:	**for** every k **do**
5:	Apply the CHM classifier C_S,L_ to the k-th tile
6:	**end for**
7:	Stitch all k tiles together to yield the probability map, M_j_
8:	Normalize M_j_
9:	Classify M_j_ using Otsu's multi-level method with G gray levels, yielding O_j_
10:	Threshold O_j_ at the G-th level, giving the initial position mask K_j_
11:	Perform morphological shrinking on K_j_
12:	Segment M_j_ by evolving active contours at initial positions specified by each unique 2D connected component of K_j_.
	Iterate α times with a smoothing factor of λ. The output is SEG_j_, the final segmentation of I_j_.
13:	**end for**

#### Binarization of probability maps

**Figure 3:**
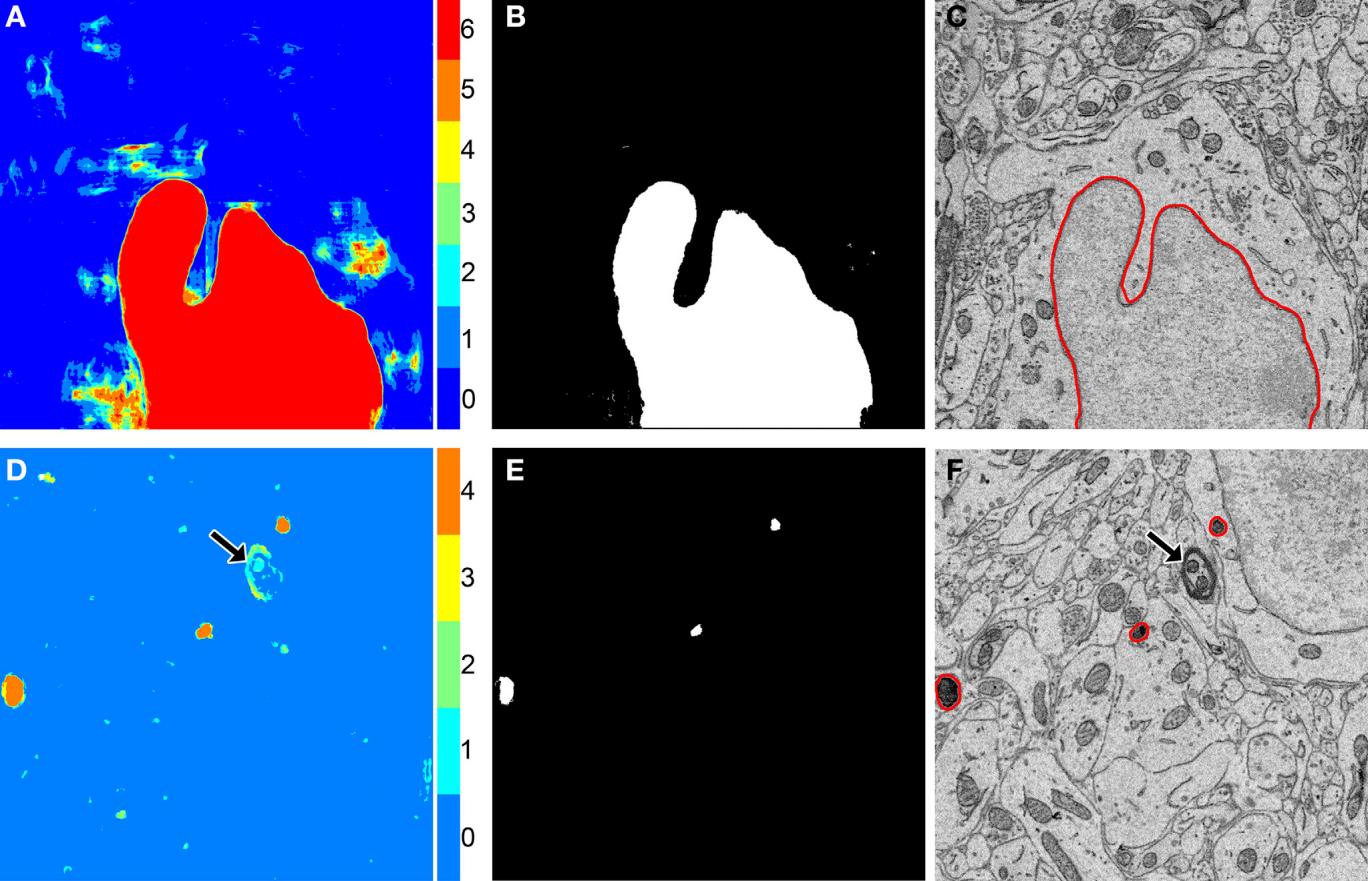
**The binarization of probability maps using active contours initialized by a multi-level Otsu threshold yields accurate segmentation results**. Colorized maps, M, of a nucleus **(A)** and lysosomes **(D)** generated by applying Otsu's method with multiple levels to probability maps obtained by CHM pixel classification. Each color corresponds to a unique level of the threshold. Six gray levels (*G* = 6) were used for the nucleus and four (*G* = 4) were used for the lysosomes. Initial positions **(B,E)** were determined by selecting pixels corresponding to only the highest levels of each threshold followed by two iterations of morphological shrinking. Output segmentations **(C,F)** were obtained by evolving active contours about each of the initial positions in **(B,E)** with 100 iterations and a smoothing factor of 8 (α = 100, λ = 8). In the case of the lysosome images, note that a myelinated axon that was originally detected by the classifier as a false positive (**D**, arrow) has been removed from the final segmentation by the application of our method (**F**, arrow).

Each probability map, M_j_, is binarized by evolving active contours (Chan and Vese, 2001) at automatically determined initial positions. For an unsupervised determination of the initial positions, the probability map M_j_ is first thresholded using Otsu's multi-level method (Otsu, 1979) with G unique gray levels (step 9 of Algorithm 1). The output from this operation is O_j_, a map in which each pixel of M_j_ has been classified into one of G unique levels, with the zeroth level corresponding to the approximate background. This map is then binarized by thresholding O_j_ at a pixel intensity of G, yielding a mask of initial positions, K_j_ (step 10 of Algorithm 1). This binary mask is then made smaller by applying two iterations of morphological shrinking (step 11 of Algorithm 1) and used to initialize the evolution of active contours with a number of iterations and smoothing factor specified by α and λ, respectively (step 12 of Algorithm 1). Each 2D connected component of K_j_ serves as a unique initial position for contour evolution. For best results, α should be at least 50. The choice of λ depends largely on the organelle target and pixel size of the test images, but in general should fall in the range of 0–8. Larger values of λ can be used when the pixel size is small. If the pixel size is too large (i.e., above 10 nm/pixel), smoothing should be turned off by setting λ to zero. The value of G significantly alters the results, and its choice is dependent on the goals of the experimenter. Low values of G tend to emphasize true positives at the risk of retaining false positives. As G is increased, false positives are more readily removed, but so are true positives. The final output from this process is SEG_j_, the organelle segmentation of the input grayscale image, I_j_. An illustration of this process is shown for two test images in Figure 3.

#### Meshing

Each output SEG_j_ is converted to the MRC format and appended to an MRC stack. Contours are drawn around each 2D connected component using the IMOD program *imodauto*. The output contours are then three-dimensionally meshed together using the program *imodmesh*, and separate 3D connected components are sorted into different objects using the program *imodsortsurf*. Meshing is performed using the low resolution option to reduce the effect of translational artifacts between subsequent image slices.

### Experimental validation

#### Tissue processing, image acquisition, and preprocessing

The suprachiasmatic nucleus (SCN) of one 3-month-old, male C57BL/6J mouse was harvested and prepared for SBEM using a standard protocol (Wilke et al., 2013). The resin-embedded tissue was mounted on an aluminum specimen pin and prepared for SBEM imaging as previously described (Holcomb et al., 2013). Imaging was performed by detection of backscattered electrons (BSE) using a Zeiss Merlin scanning electron microscope equipped with a 3View ultramicrotome (Gatan). The SBEM image stack was acquired in ultrahigh vacuum mode using an accelerating voltage of 1.9 kV, a pixel dwell time of 500 ns, and a spot size of 1.0. Sectioning was performed with a cutting thickness of 30 nm. BSE images were acquired at 800x magnification with a raster size of 32,000 pixels × 24,000 pixels, yielding a pixel size of 3.899 nm/pixel. A total of 1283 serial images were acquired, resulting in an image stack with tissue dimensions of roughly 124.8 × 93.6 × 38.5 μm (~450,000 μm^3^). The specimen was then removed from the chamber, and an image of a diffraction grating replica specimen (Ted Pella, Redding, CA, U.S.A.) was acquired for calibration of the lateral pixel size. Low magnification images of the block-face were acquired before and after sectioning. Image alignment was performed as described in Section Image Alignment and Histogram Specification. Following alignment, the stack was downsampled in the XY-plane by a factor of two, yielding a final stack with pixel dimensions of 16,000 × 12,000 × 1283 and pixel sizes of 7.799 nm/pixel and 30 nm/pixel in the lateral and axial dimensions, respectively. Since preliminary results did not demonstrate noticeable differences in the output of our method between the native resolution stack and the downsampled stack, downsampling was performed to reduce processing time. Exact histogram specification was performed as previously described. All image alignment and pre-processing steps were performed on a custom workstation (Advanced HPC, San Diego, CA, U.S.A.) with the following configuration: Xeon X5690 3.47 GHZ CPU, 48 GB RAM, 32 TB HDD, NVIDIA Quadro FX 3800, CentOS release 6.2.

#### Automatic segmentation

The four types of organelles targeted for automatic segmentation were mitochondria, lysosomes, nuclei, and nucleoli. These targets were chosen because they are morphologically and texturally diverse, and thus pose a significant test of the robustness of our method.

For each organelle target, 90 seed points were placed throughout the SBEM stack as described in Section Generation of Training Images and Labels. Training data and labels were created using the values shown in Table 1. Of the 90 tiles generated for each organelle, 50 were randomly selected for use in training a CHM classifier; the other 40 were set aside to use as test data for validation. Organelle-specific CHM classifiers were trained using the values shown in Table 1. The performances of all classifiers were evaluated by preparing receiver operating characteristic (ROC) curves (Fawcett, 2006). Each classifier was then used to generate probability maps of the 40 test images corresponding to its organelle. Segmentation was performed as described in Section Binarization of Probability Maps using the values shown in Table 1. All training, pixel classification, and segmentation steps were performed on the National Biomedical Computation Resource (NBCR) cluster, rocce.ucsd.edu (http://rocce-mgr.ucsd.edu/).

**Table 1:** **Parameter sets used for the validation of specific organelle targets**.

**Parameter**	**Variable**	**Mitochondria**	**Lysosomes**	**Nuclei**	**Nucleoli**
Number of training slices	N	50	50	50	50
Lateral dimensions of each training slice	Q, R	500, 500	500, 500	500, 500	500, 500
Number of CHM levels	L	2	2	2	2
Number of CHM stages	S	2	2	2	2
Size of tile array	m, n	2, 2	2, 2	2, 2	2, 2
Tiling overlap	U	50	50	20	50
Gray levels for multi-level Otsu thresholding	G	3	2	2	2
Active contour iterations	α	80	200	300	90
Smoothing factor	λ	7	4	8	10

#### Validation of the active contour segmentation of CHM probability maps

Evaluation metrics were computed for each set of organelle-specific test images by comparing their segmentations with manually segmented ground truth. For each stack, the confusion matrix consisting of the number of true positive (TP), false positive (FP), true negative (TN), and false negative (FN) pixels was computed and used to calculate the true positive rate (TPR), false positive rate (FPR), precision, accuracy, and *F*-value, such that:

$$\begin{array}{l} \text{TPR}=\frac{\text{TP}}{\text{TP+FN}}\\ \text{FPR}=\frac{\text{FP}}{\text{FP+TN}}\\ \text{Precision}=\frac{\text{TP}}{\text{TP+FP}}\\ \text{Accuracy}=\frac{\text{TP+TN}}{\text{TP+FN+FP+TN}}\\ \text{F-value}=\frac{\text{2 }\times \text{ Precision }\times \text{ TPR}}{\text{Precision+TPR}} \end{array}$$

This analysis was then repeated with segmentations generated from the same probability maps, but with a number of different unsupervised binarization algorithms: (1) Minimum error thresholding (Kittler and Illingworth, 1986), (2) Maximum entropy thresholding (Kapur et al., 1985), and (3) Otsu's single-level method (Otsu, 1979). The performance of each algorithm, as quantified with the above metrics, was compared against that of our own method for each organelle target.

Since ground truth was available, the pixel intensity threshold that maximized the *F*-value of each probability map with respect to its corresponding ground truth was determined by computing the *F*-value at incrementally increasing thresholds from [0, …, 1] and taking the maximum value.

### Scale-up to teravoxel-sized datasets

#### Determination of optimal downsampling levels for different organelles

Since the segmentation of entire SBEM datasets is computationally intensive, we first decided to determine to what degree input images could be downsampled before segmentation results were adversely affected. Downsampled versions of each set of training images, training labels, and test images were prepared for all four organelle targets. Downsampling was performed by factors of two, three, four, and five, yielding pixel sizes of roughly 15.59, 23.39, 31.19, and 38.90 nm/pixel, respectively. CHM classifiers with two stages and two levels were trained for each set of downsampled, organelle-specific training images and labels. Probability maps were computed with *m* = 2, *n* = 2, and *U* = 20. Segmentations were generated using the active contour method with *G* = 2, α = 100, and λ = 0. For each set of output segmentations, evaluation metrics were computed as described in Section Validation of the Active Contour Segmentation of CHM Probability Maps.

#### Segmentation of organelles from a full SBEM stack

The entire test dataset was laterally downsampled by a factor of eight, yielding a final stack with dimensions of 4000 × 3000 × 1283 pixels. The corresponding CHM classifiers generated in Section Determination of Optimal Downsampling Levels for Different Organelles were applied to produce stacks of probability maps at this pixel size for nuclei, nucleoli, and mitochondria. Processing was performed using an 8 × 6 tile array with an overlap of 20 pixels between adjacent tiles. Tiling, pixel classification, stitching, and binarization were performed using one CPU for each input image. One hundred total CPUs were used, such that 100 images were processed in parallel to expedite processing. All steps were performed on the National Biomedical Computation Resource (NBCR) cluster, rocce.ucsd.edu. Following probability map generation, all images were appended to organelle-specific MRC stacks, and contours and surface renderings were generated as described in Section Meshing.

### Comparison to a previously published algorithm

The results of our approach to nuclear automatic segmentation were validated by comparison with the results obtained by the algorithm of Tek et al. (2014). The full dataset was first downsampled to isotropic voxel dimensions (30 × 30 × 30 nm), resulting in a stack of size 4029 × 3120 × 1283 voxels. Training data and images consisted of a 500 × 500 × 50 subvolume of the downsampled stack containing two adjacent nuclei. Ground truth data were generated by manual segmentation of all neuronal, glial, and endothelial cell nuclei across fifty consecutive slices from the center of the dataset. A CHM pixel classifier with two stages and two levels was trained and applied to all images in the stack. Similarly, an ilastik voxel classifier was trained using all possible features with the same training images serving as input (Sommer et al., 2011). This classifier was subsequently applied to all images in the downsampled stack. CHM probability maps were binarized using the proposed method. The ilastik probability maps were binarized by thresholding at the level *p* = 0.5, followed by the application of the object detection algorithm of Tek and colleagues with V_th1_ and V_th2_ set to 25 and 10,000, respectively (Tek et al., 2014).

The source code for CHM and all related scripts are available to download from http://www.sci.utah.edu/software/chm.html. The training images, training labels, and test images used in this study have also been made available to download at this URL.

## Results

**Figure 4:**
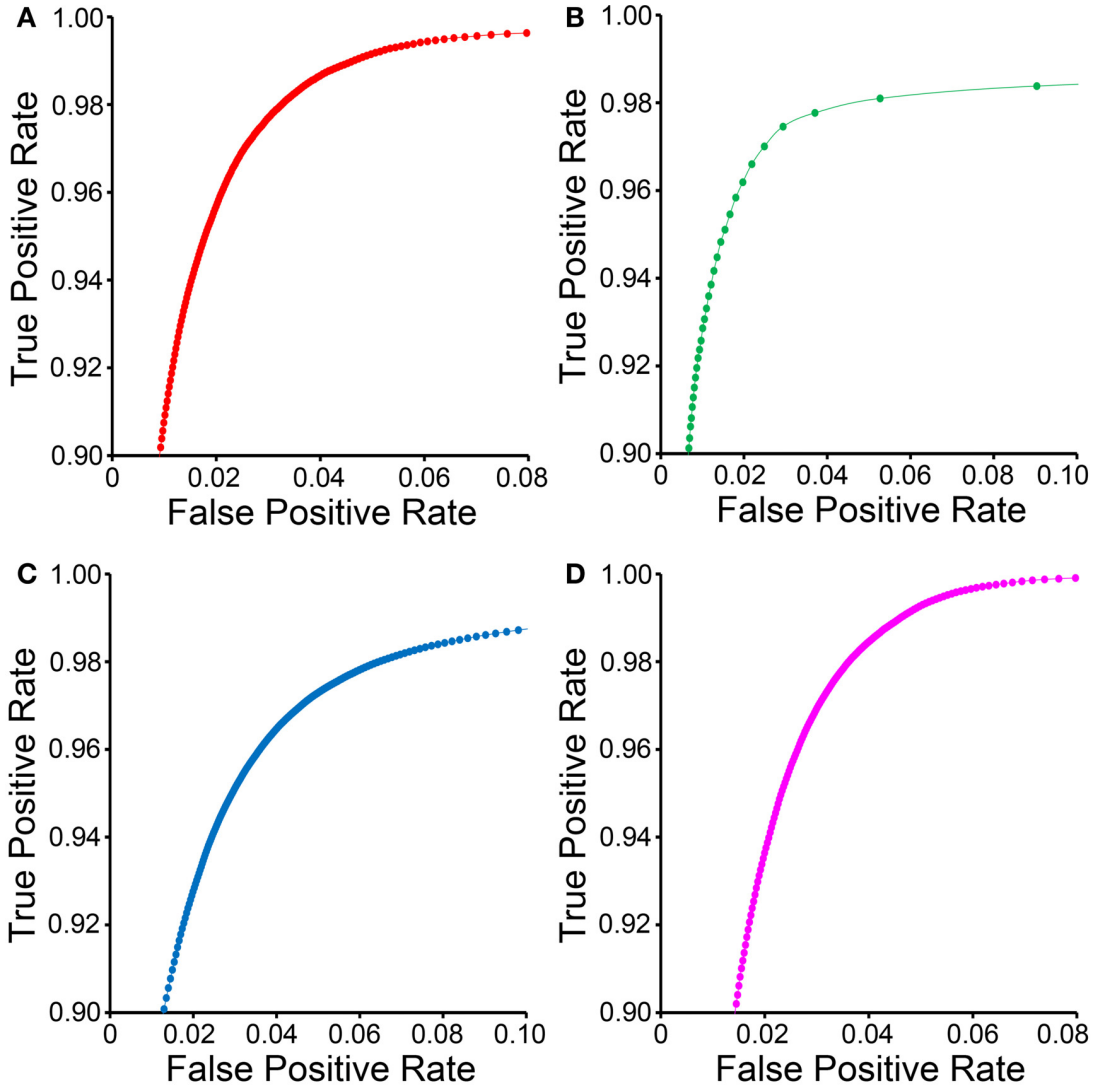
**ROC curves for CHM classifiers of various organelles**. ROC curves for mitochondrial **(A)**, lysosomal **(B)**, nuclear **(C)**, and nucleolar **(D)** CHM classifiers generated with two stages and two levels.

ROC curves for each organelle-specific CHM classifier are shown in Figure 4. In comparison to those for the other organelle classifiers, the ROC curve for the lysosomal classifier (Figure 4B) demonstrates a sparseness of data points with a low FPR. This is due to the extreme electron density of the lysosomal compartment and the number of other features in EM images that closely approximate it. Myelin sheaths (Figure 3D), plasma membranes, and other organelles cut *en face* can resemble the lysosomal compartment in both pixel intensity and texture and are frequently detected as false positives. Therefore, intelligent post-processing routines that utilize size and morphology are needed to separate lysosomes from such false positives.

**Figure 5:**
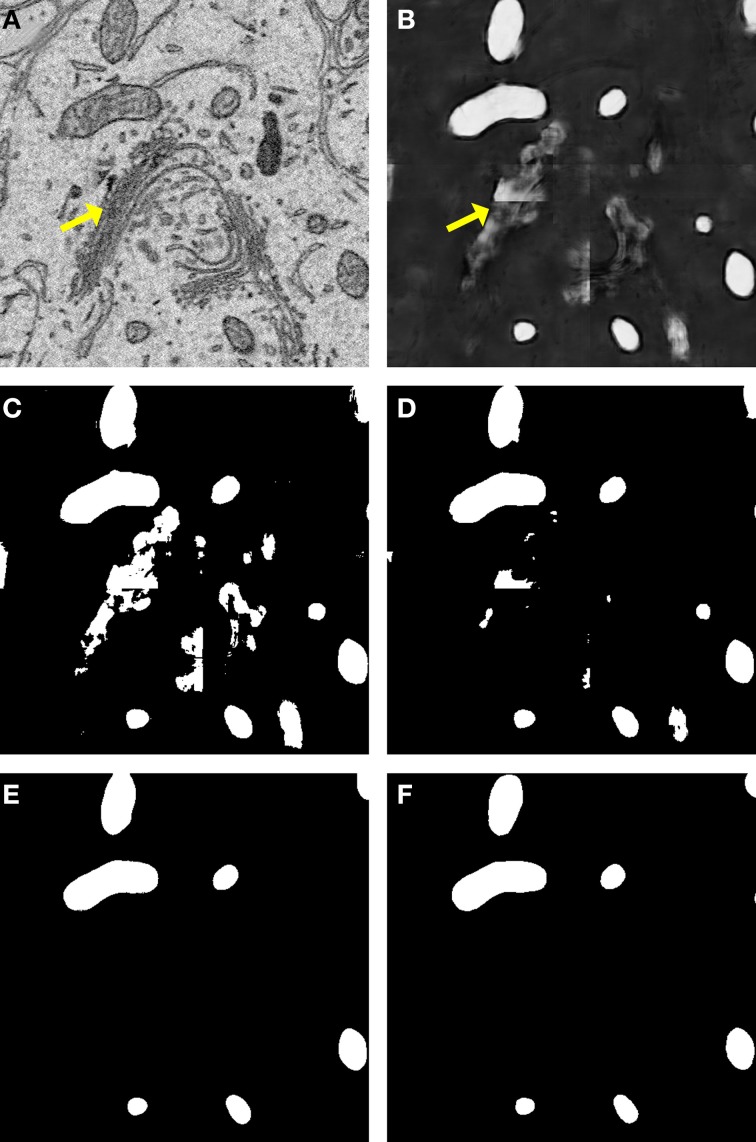
**Binarization of probability maps using active contours outperforms other methods**. A CHM classifier for mitochondria was applied to a 500 × 500 pixel test image **(A)**, generating the probability map shown in **(B)**. Note that regions of pixels corresponding to the Golgi apparatus (yellow arrows) were detected in the probability map. The Golgi apparatus can often confuse mitochondrial pixel classifiers because it has a texture very similar to that of the mitochondrial matrix. The results of binarization of the probability map using maximum entropy **(C)** and Otsu's single-level method **(D)** are shown. Using these techniques, regions of the Golgi are permitted into the final segmentation as false positives. The resultant segmentation obtained by our method of binarization with active contours (*G* = 2, α = 100, λ = 8) is shown in **(E)**. Instances of the Golgi apparatus were automatically removed during processing. This segmentation (*F* = 0.863, accuracy = 0.985) is a highly faithful representation of the ground truth **(F)**.

**Figure 6:**
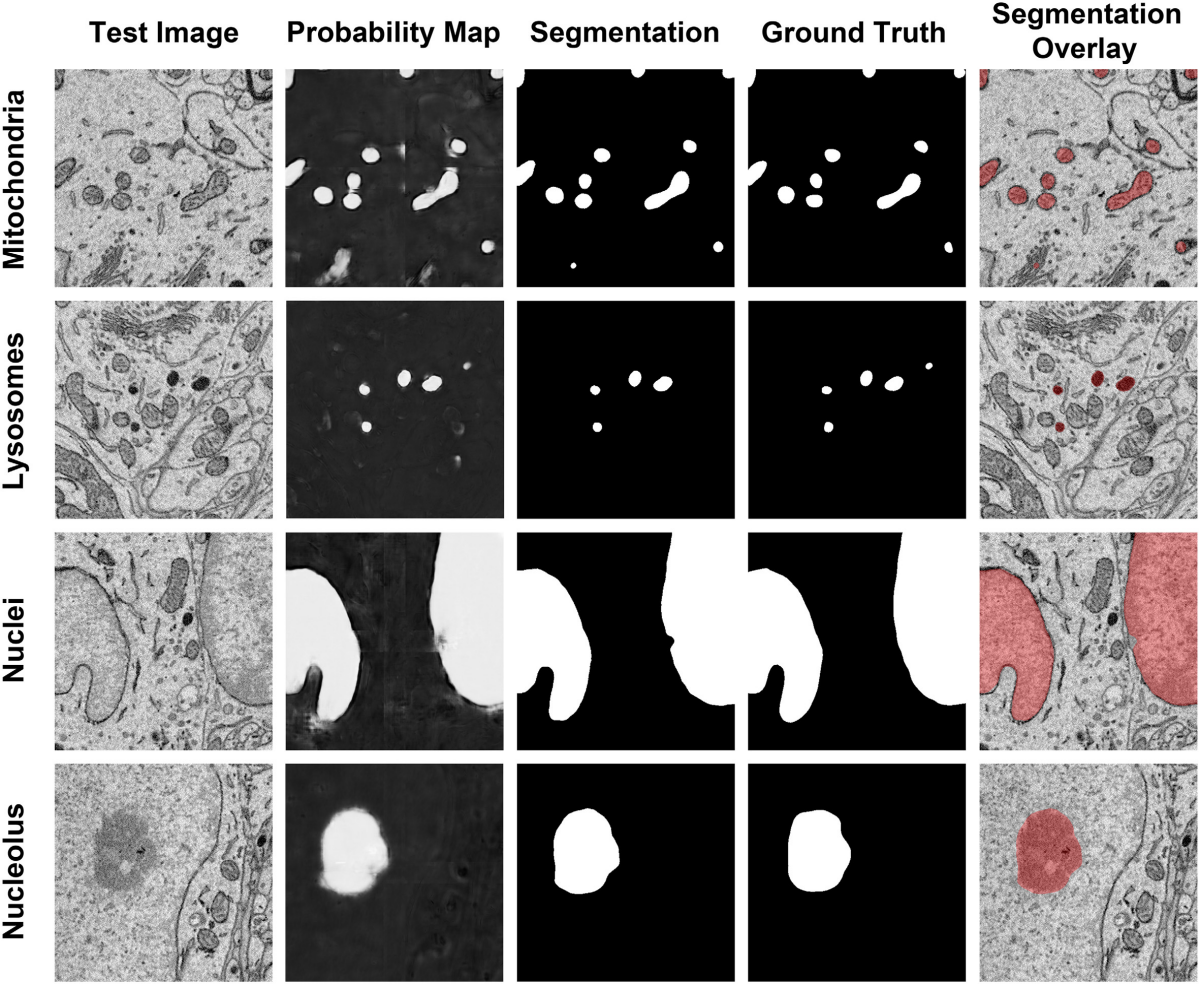
**The results of our method are consistent when applied to diverse organelle targets**. The application of our method to different organelle targets yields consistent results without the need to significantly change the input parameters. Shown here are test images, each of size 500 × 500 pixels, and their corresponding probability maps, segmentations, and manually segmented ground truth images. The final column shows a transparent overlay of the segmentation onto the test image. The evaluation metrics for each test image are as follows: Mitochondria, *F* = 0.844, accuracy = 0.984; lysosomes, *F* = 0.872, accuracy = 0.997; nuclei, *F* = 0.971, accuracy = 0.971; nucleoli, *F* = 0.91, accuracy = 0.977.

A comparison of our proposed active contour binarization method to the other methods tested is shown in Figure 5 using mitochondria as an example. Since the Golgi apparatus can sometimes display a texture similar to that of the mitochondrial matrix, the presence of this organelle can confuse the mitochondrial classifier (Figures 5A,B, arrows). Segmentations generated with the maximum entropy algorithm (Figure 5C, recall = 0.992, precision = 0.498, *F* = 0.670, accuracy = 0.948) and Otsu's single-level method (Figure 5D, recall = 0.958, precision = 0.687, *F* = 0.812, accuracy = 0.977) retain elements of the Golgi apparatus as false positives. However, probability map binarization using the proposed active contour method eliminates these false positives (Figure 5D, recall = 0.908, precision = 0.804, *F* = 0.863, accuracy = 0.985) when compared to the ground truth (Figure 5E). Output probability maps and active contour segmentations from example test images of each organelle are shown in comparison to their corresponding ground truth in Figure 6.

The segmentation evaluation metrics for each full stack of 40 organelle-specific test images are shown in Table 2. The proposed active contour segmentation method resulted in a superior recall for all four organelles and a superior *F*-value for mitochondria, lysosomes, and nucleoli when compared to the other segmentation methods. The *F*-value for nuclear segmentation is negligibly better using Otsu's single-level method. The lack of distinction between these two binarization methods for nuclei is due largely to the already high quality of nuclear probability maps. The accuracy values obtained for each stack using active contour segmentation were 0.985, 0.997, 0.972, and 0.979 for mitochondria, lysosomes, nuclei, and nucleoli, respectively.

**Table 2:** **Segmentation evaluation metrics for the tested organelle targets using various methods of probability map binarization**.

	***F*-value**	**Precision**	**Recall**	**Jaccard Index**
**MITOCHONDRIA**				
Minimum Error	0.635	0.994	0.466	–
Max. Entropy	0.669	0.991	0.505	–
Otsu Single-level	0.816	0.957	0.712	–
Active Contours	0.877	0.867	0.886	0.780
**LYSOSOMES**				
Minimum Error	0.433	0.985	0.277	–
Max. Entropy	0.492	0.940	0.508	–
Otsu Single-level	0.812	0.899	0.737	–
Active Contours	0.841	0.854	0.828	0.726
**NUCLEI**				
Minimum Error	0.963	0.958	0.968	–
Max. Entropy	0.644	0.603	0.692	–
Otsu Single-level	0.971	0.979	0.963	–
Active Contours	0.970	0.973	0.968	0.942
**NUCLEOLI**				
Minimum Error	0.781	0.998	0.641	–
Max. Entropy	0.811	0.996	0.684	–
Otsu Single-level	0.898	0.973	0.835	–
Active Contours	0.910	0.902	0.918	0.835

**Figure 7:**
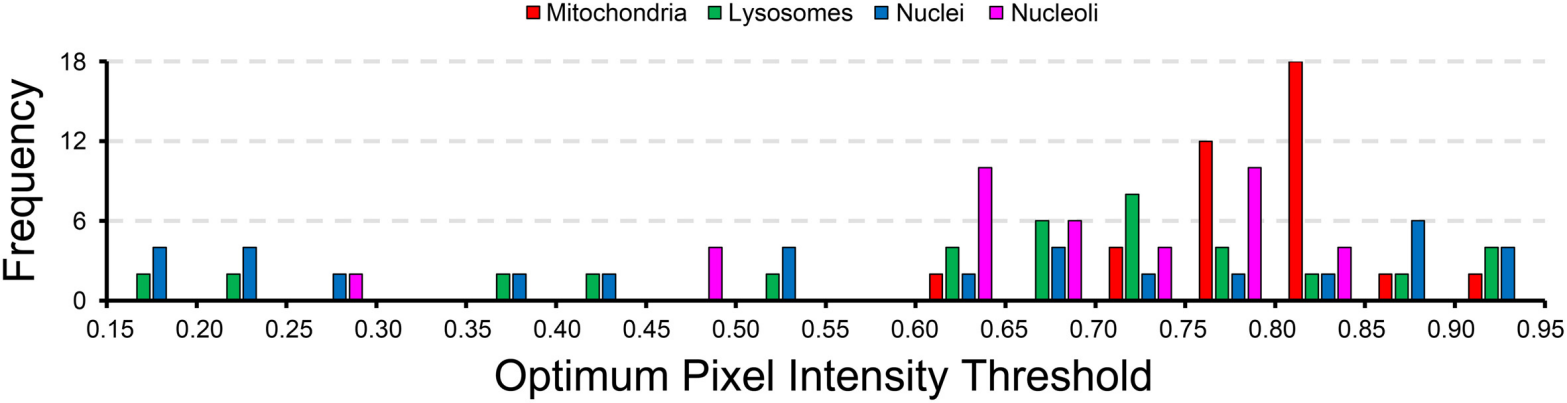
**The wide distribution of optimum pixel intensity thresholds demonstrates the usefulness of our method for probability map binarization**. The probability map pixel intensity threshold that maximized the *F*-value with respect to ground truth was determined for all of the 40 test images analyzed for each organelle. The histogram of optimal thresholds shown here demonstrates the need for an unsupervised method of binarization. Simple thresholding of all probability maps at a single user-specified intensity level would result in poor results for many of these test images. Binarization using our method circumvents this problem by adapting the results to the unique histogram of each probability map in an unsupervised manner.

A histogram of the probability map pixel intensity thresholds that maximize the *F*-value for each test image are show in Figure 7. The wide spread of optimal threshold values for each organelle demonstrates the importance of using an unsupervised algorithm for probability map binarization, such as the one proposed here. Simply setting a pixel intensity threshold for each probability map would yield poor segmentations for a number of test images. This is especially true in very large SBEM images, where alterations in staining or focus may occur differentially throughout regions of the image stack.

**Figure 8:**
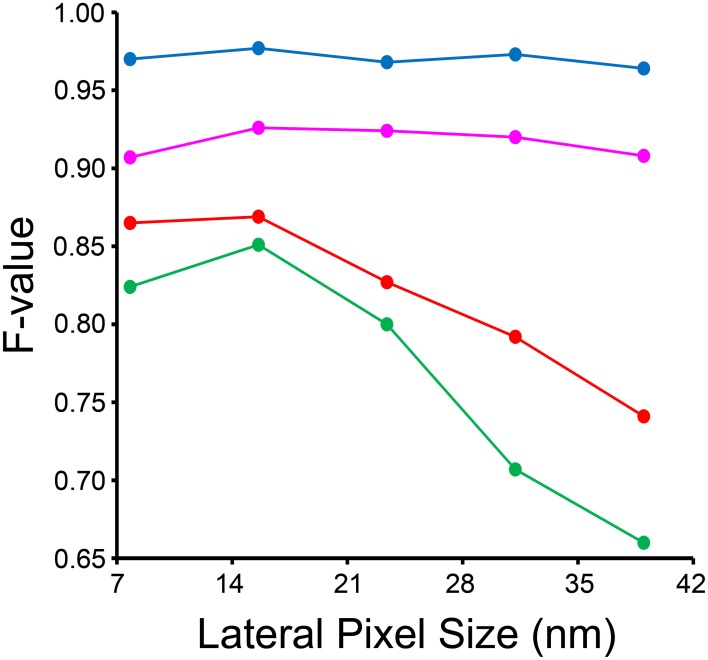
**Input images can be downsampled to various degrees before the segmentation results are negatively affected**. Each organelle-specific stack was downsampled by factors of two, four, six, eight, and ten. Separate classifiers were trained at each different pixel size and segmentations were generated for each stack using our method. Here, the *F*-value of each resultant stack is compared across the different pixel sizes obtained after downsampling. The *F*-value of nuclei (blue) and nucleoli (magenta) is remarkably independent of the level of downsampling across all levels tested. The *F*-values for mitochondria (red) and lysosomes (green) significantly decline as the level of downsampling is increased.

The results of our downsampling experiment are shown in Figure 8. The resultant *F*-value for segmentation of nuclei and nucleoli remains remarkably consistent across the whole range of pixel sizes tested. The *F*-values for mitochondria and lysosomes exhibit substantial reductions at pixel sizes greater than ~15 nm/pixel, corresponding to an overall downsampling of the original SBEM stack by a factor of four. The persistence of a high *F*-value across all scales tested for nuclei and nucleoli is likely due to their larger size and more regular texture in comparison to the other organelles. This is especially true for mitochondria, whose cristae architectures may differ dramatically from region to region.

The required wall clock time and random access memory (RAM) required for CHM classifier training and pixel classification for each organelle at each level of downsampling are shown in Table 3. The time and RAM required for probability map binarization are not shown because they are negligible with respect to training and classification. These results indicate that, in cases where segmentation accuracy is not dramatically affected, a vast amount of time and computational resources can be saved by downsampling the input image stacks. Simple extrapolation of pixel classification times shows that the time required by a single CPU to apply a nuclear pixel classifier to our full test dataset would be reduced from ~5.9 to ~0.4 years when the input data are downsampled by a factor of 10.

**Table 3:** **Runtime and memory requirements for nuclear CHM classifier training and pixel classification at various levels of downsampling**.

**nm/pixel**	**Classifier Training**			**Pixel Classification**	
	**Dimensions**	**Time (h)**	**RAM (GB)**	**Time (min)**	**RAM (GB)**
7.79	500 × 500 × 50	23.98	87.24	12.73 ± 0.90	4.54 ± 0.03
15.59	250 × 250 × 50	20.35	39.38	4.67 ± 0.15	2.08 ± 0.04
23.39	166 × 166 × 50	7.95	18.16	2.03 ± 0.03	1.68 ± 0.05
31.19	125 × 125 × 50	4.71	10.83	1.18 ± 0.02	1.52 ± 0.04
38.90	100 × 100 × 50	3.18	7.38	0.90 ± 0.04	1.41 ± 0.04

The dimensions of the stack of training images and labels used to train the classifier are given. The values for pixel classification correspond to the average values required to generate a probability map for one tile of roughly 60 μm^*2*^ at the tissue level (1000 × 1000 pixels at 2x downsampling). Values are reported as the mean and standard deviation (N = 40 for each). Time is reported as the wall clock time for the indicated process.

**Figure 9:**
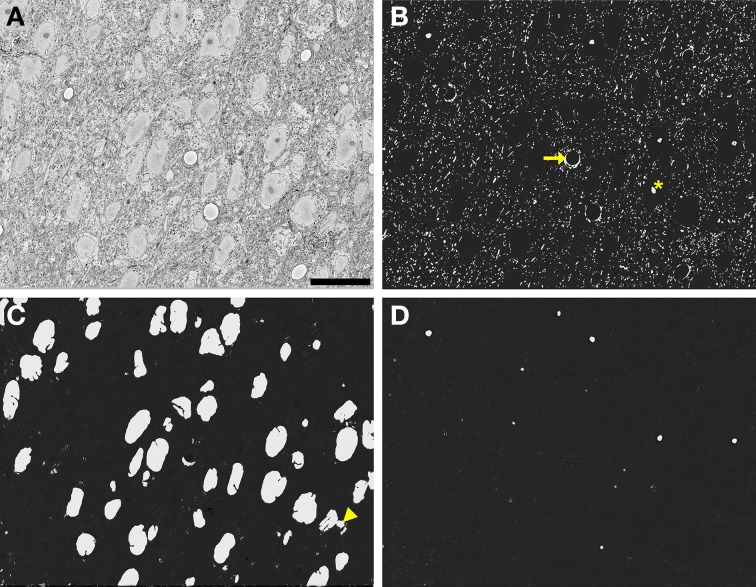
**Automatic segmentation can be efficiently scaled to handle full slices from teravoxel-sized SBEM datasets**. Probability maps of full images from the SCN dataset were generated by downsampling the image, computing probability maps of individual tiles, and stitching these tiled maps together. Shown here are probability maps of mitochondria **(B)**, nuclei **(C)**, and nucleoli **(D)** computed from the same full slice **(A)**. The full slice was downsampled by a factor of two prior to mitochondrial pixel classification and a factor of eight before nuclear and nucleolar pixel classification. Common residual errors during mitochondrial pixel classification are the false detection of endothelial cells (arrow) and nucleoli or clusters of chromatin in the nucleus (asterisk). A common error encountered during nuclear pixel classification is the false detection or regions of cytoplasm devoid of membrane-bound organelles (arrowhead). These residuals are frequently removed by the application of the proposed probability map segmentation algorithm. Scale bar = 20 μm.

These time and memory requirements were dramatically reduced by implementing tiling and processing over multiple CPUs. During segmentation of the full, downsampled dataset, the average processing time per 500 × 500 tile was 3.28 ± 0.39 min (average and standard deviation, *N* = 600), with no significant difference in average time between organelles. By utilizing parallel processing with 100 CPUs, probability maps for the entire stack were generated in roughly 33 h. An example full slice and its corresponding nuclear probability map are shown in Figures 9A,C. Figures 9B,D depict additional probability maps of mitochondria and nucleoli, respectively. The full slice probability maps of these other organelles were computed in a manner similar to that of the nuclei.

When applied to the segmentation of nuclei from the full SCN dataset following downsampling to isotropic voxel dimensions, the proposed method achieved a precision, recall, and *F*-value of 0.976, 0.977, and 0.977, respectively. Similarly, the method of Tek et al. (2014) achieved a precision, recall, and *F*-value of 0.976, 0.542, and 0.697, respectively, when applied to the same dataset using the same training data. Due to an already high precision and low number of false positives, the final object classification step performed by Tek and coworkers was omitted. Evaluation metrics were computed using fifty consecutive manually annotated slices as ground truth.

**Figure 10:**
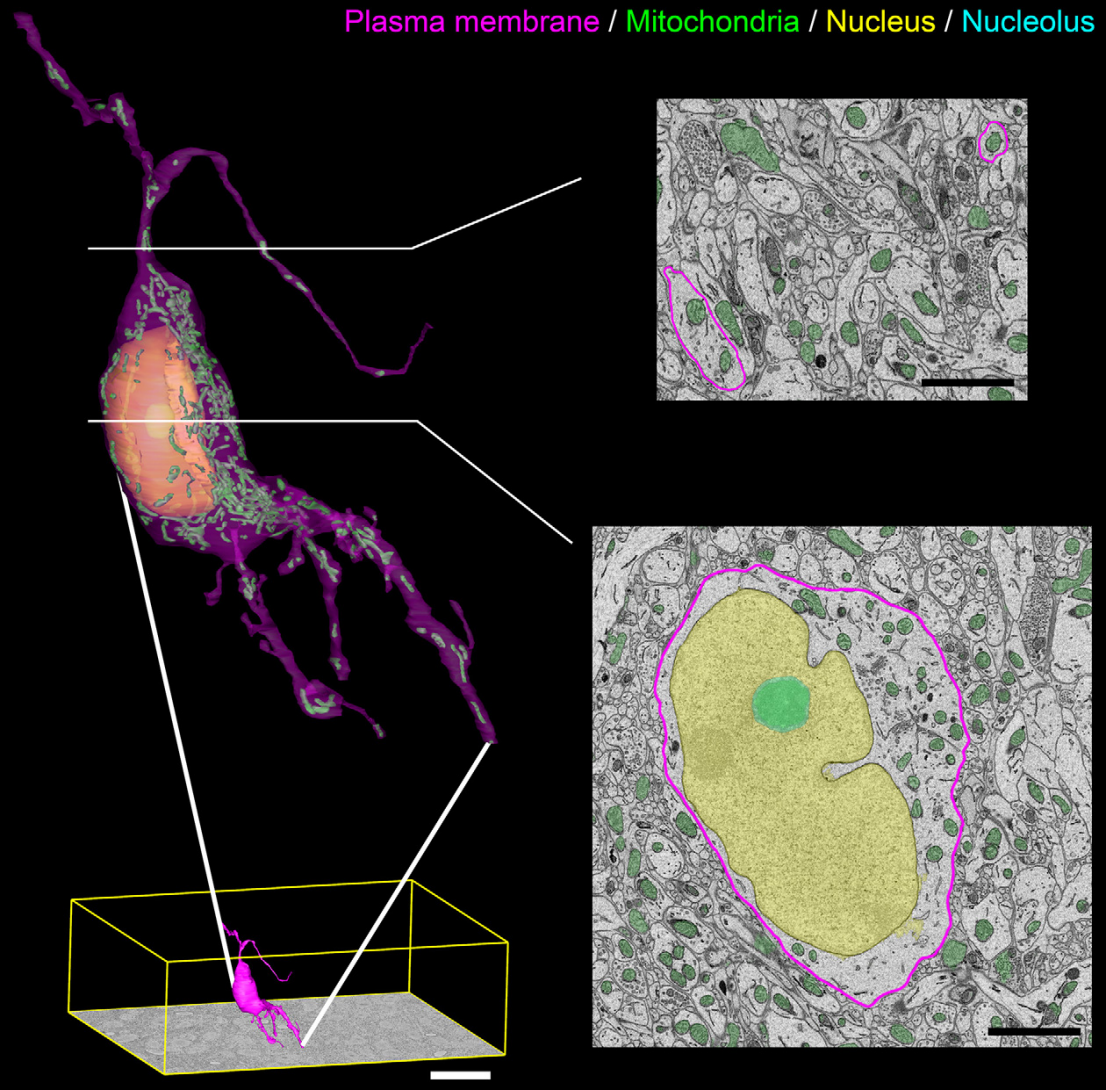
**Output surface renderings of manually segmented organelles within an SCN neuron**. The plasma membrane of a neuron was manually traced in its entirety throughout the dataset. The size of this neuron with respect to the full dataset (bottom left, scale bar = 20 μm) demonstrates the scale of the segmentation challenge. An enlarged version of this neuron with a transparent plasma membrane is shown in the upper left corner. Surface renderings of the nucleus (yellow), nucleolus (cyan), and mitochondria (green) were generated from the output of our automatic segmentation workflow. Two cross-sectional planes through the neuron reveal the corresponding SBEM slice with transparent overlays of the probability maps for the three organelles (scale bar = 2 μm). Output renderings such as these can be used to analyze any number of parameters, including organelle morphology and clustering throughout the whole cell.

A surface rendering of a full SCN neuron containing renderings of its nucleus, nucleolus, and mitochondria is shown in Figure 10. The plasma membrane of the neuron was manually segmented by a trained neuroanatomist. The surface renderings of all organelles were automatically generated, with minor manual corrections applied.

## Discussion

As recently as a few years ago, the notion of reconstructing and morphologically characterizing the organelle networks of even a few whole cells was considered a monumental challenge (Noske et al., 2008). The advent and widespread adoption of high throughput, volumetric EM techniques has threatened to change that notion, with the caveat that our ability to segment and analyze data must first catch up with our ability to collect it. With that goal in mind, this study aimed to develop a method for the accurate automatic segmentation of organelles in EM image stacks that: (1) could be easily adapted to any organelle of interest, and (2) could be applied to teravoxel-sized datasets in a computationally efficient manner.

Since it does not make any large-scale, *a priori* assumptions about the morphology of the segmentation target, the proposed method can be applied to segment diverse organelles with ease. The only geometrical properties assumed throughout the method are boundary smoothness and a cross-sectional area that is sufficient enough to prevent the removal of true positives following binary shrinking. Both of these assumptions are valid for virtually all organelles under practical imaging conditions. CHM classifiers can be trained for any dataset or organelle target if given the proper training data, and the output segmentations from our method can be tuned to the demands of unique experiments. For example, decreasing the number of gray levels, G, used in the multi-level Otsu thresholding step will emphasize true positives at the expense of including false positives, which can often be excluded by post-processing filters. Additionally, it is easier to remove false positives by manual correction or crowd-sourcing (Giuly et al., 2013) than it is to add missing true positives.

The proposed method performed favorably when compared to a recently published algorithm for the automatic segmentation of cell nuclei (Tek et al., 2014). It is interesting to note that the performance of our method was very similar when trained using either images from consecutive slices of the same nuclei (precision = 0.976, recall = 0.977) or single slice images from a variety of nuclei (precision = 0.973, recall = 0.968). This similarity demonstrates the robustness of the CHM pixel classifier for this task. It is likely that the segmentation results obtained by applying the method of Tek and colleagues to the SCN dataset could be strengthened by training an ilastik voxel classifier against a greater diversity of nuclei.

Another advantage of the proposed method lies in its scalability to full datasets. The generation of probability maps from small tiles of the input image minimizes the required RAM. Additionally, it allows for computation to be easily expedited by parallelizing the processing of individual tiles across multiple CPUs. Our demonstration that accurate results for certain organelles can be achieved on downsampled stacks also helps expedite processing. One can envision an experiment in which a teravoxel-sized SBEM stack collected at high resolution for axon tracking can then be downsampled and have its nuclei or mitochondria automatically segmented at a fraction of the computational cost that would have been required at its native resolution. As innovative methods to rapidly acquire even larger datasets continue to be developed (Mohammadi-Gheidari and Kruit, 2011; Helmstaedter et al., 2013; Marx, 2013), this reduction in computational cost will prove critical.

Although it is beyond the scope of this paper, a number of 3D post-processing steps that would lead to further improvements in the results of automatic segmentation can be proposed. A simple size exclusion filter could be applied to 3D connected components to remove false positives that do not fall within the possible size range for the given organelle. A scan over every segmented slice of each 3D component could be performed to look for aberrant spikes or troughs in 2D metrics such as perimeter or area. The locations of these spikes and troughs would indicate slices on which a poor segmentation occurred, and these slices could be correspondingly removed and replaced by interslice interpolations. The application of such processes to the output from our method will be the subject of future development.

In conclusion, this paper introduces novel methods for the automatic segmentation of organelles from EM image stacks that are both robust and able to handle datasets of any size. These tools fill a critical need by allowing for the quantitative analysis of volumetric EM datasets at a scale between that of current connectomics approaches (Briggman and Denk, 2006; Anderson et al., 2011; Bock et al., 2011; Briggman et al., 2011; Kleinfeld et al., 2011; Varshney et al., 2011; Helmstaedter et al., 2013; Kim et al., 2014) and that afforded by genetically encoded markers for small molecule localization (Shu et al., 2011; Martell et al., 2012; Boassa et al., 2013).

## Authors and contributors

Alex J. Perez, Mojtaba Seyedhosseini, Tolga Tasdizen, Satchidananda Panda, and Mark H. Ellisman designed research. Alex J. Perez, Mojtaba Seyedhosseini, Thomas J. Deerinck, and Eric A. Bushong performed research. Alex J. Perez and Mojtaba Seyedhosseini analyzed data. Alex J. Perez wrote the paper.

### Conflict of interest statement

The authors declare that the research was conducted in the absence of any commercial or financial relationships that could be construed as a potential conflict of interest.
